# One Stop Management in Acute Stroke: First Mothership Patient Transported Directly to the Angiography Suite

**DOI:** 10.1007/s00062-017-0574-z

**Published:** 2017-03-07

**Authors:** Marios-Nikos Psychogios, Mathias Bähr, Jan Liman, Michael Knauth

**Affiliations:** 1 Department of Neuroradiology, University Medical Center Göttingen, Robert-Koch-Str. 40, 37075 Göttingen, Germany; 2 Department of Neurology, University Medical Center Göttingen, Robert-Koch-Str. 40, 37075 Göttingen, Germany

## Introduction

Endovascular treatment (EVT) of acute ischemic stroke is the new standard of treatment in patients with large artery occlusion (LAO) [1, 2]. A short time from symptom onset to reperfusion and especially from hospital admission to reperfusion is crucial for a good clinical outcome [3]. To reduce the time from hospital admission to reperfusion, we combined imaging and treatment in the angiography suite. This case report describes a one stop management approach of depicting acute ischemic signs and excluding intracranial hemorrhage with nonenhanced flat detector computed tomography (FDCT), identifying large vessel occlusion on a multiphase flat detector CT angiography (mpFDCTA) and treating the patient with endovascular thrombectomy, all in the same room.

## Case Report

**Fig. 1:**
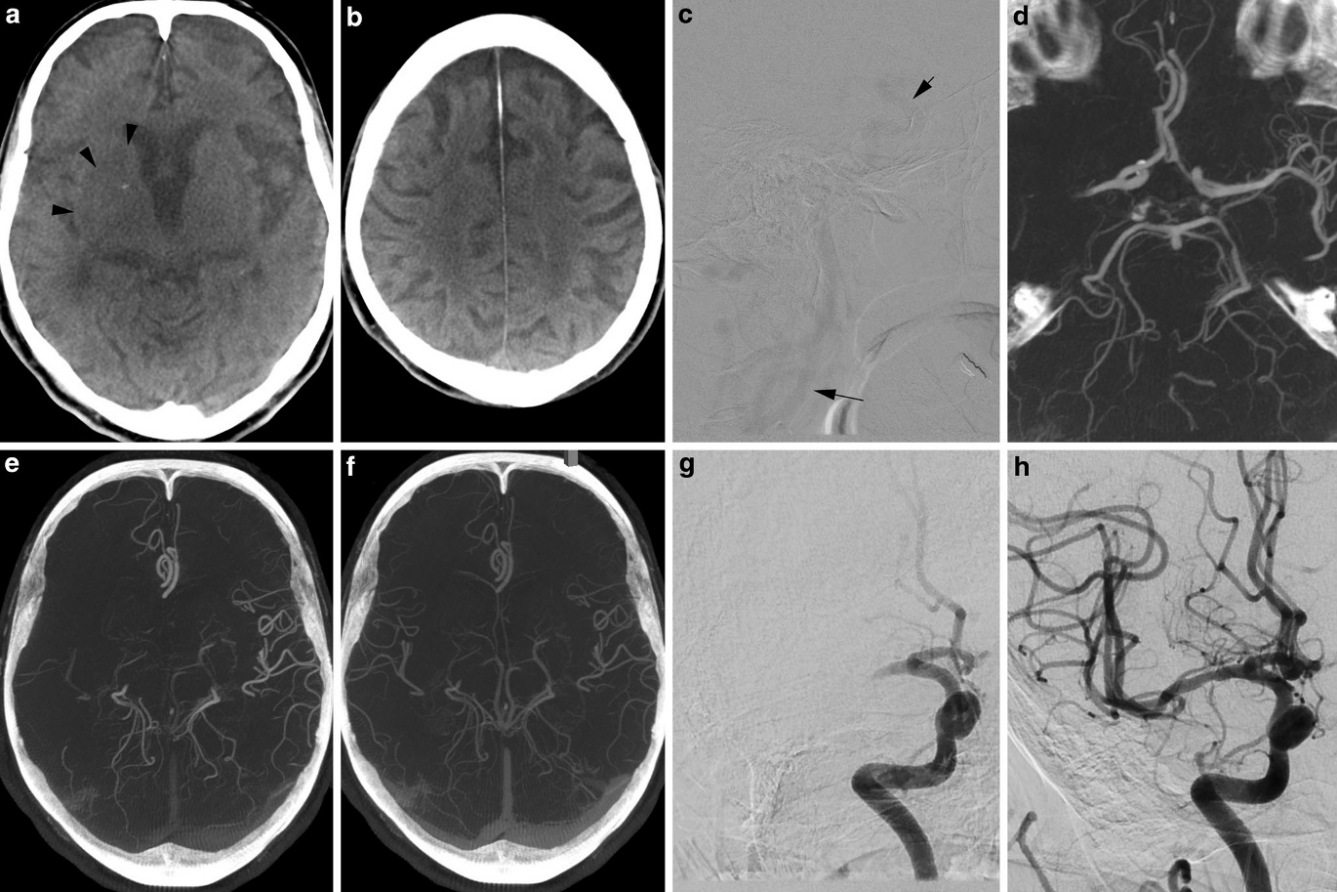
**a**, **b** show FDCT images of an 89-year-old female patient. An intracerebral hemorrhage can be excluded. Hypodensities (**a** *arrowheads*) of the right insula, caudate and lentiform nucleus result in an ASPECTS of 7. **c** We used digital subtraction angiography (DSA) to time the first phase of the mpFDCTA and started with the rotation after intravenously injecting contrast media and seeing enhancement of the distal carotid artery (**c** *arrows*). Reconstruction of the arterial mpFDCTA phase shows a proximal middle cerebral artery thrombosis on the right side (**d**) and just a few visible pial vessels within the occluded vascular territory (**e**). Note the very good depiction of even small intracranial vessels (**c** e. g. the distal superior cerebellar artery) due to the technical features of the mpFDCTA acquisition. The second (venous) mpFDCTA phase (**f**) delineates delayed filling in of peripheral vessels with decreased prominence and extent as well as ischemic regions with no vessels. DSA images show the persistent proximal occlusion before (**g**) and the successful recanalization of the middle cerebral artery (**h**) after thrombectomy with the SAVE technique [4] (symptom to door time: 42 min, door to FDCT time: 8 min, door to groin time: 23 min, groin to reperfusion: 36 min, door to reperfusion: 59 min, symptom to reperfusion: 101 min)

An 89-year-old female patient was admitted to our hospital with symptoms suggestive of stroke 42 min after symptom onset. A brief neurological examination on arrival showed a National Institutes of Health stroke scale (NIHSS) of 19 and a modified Rankin scale of 5. The patient was directly transferred to the angiography suite (Artis Q; Siemens Healthcare, Forchheim, Germany) where FDCT and mpFDCTA images were acquired. A standard 20 s noncontrast rotational dataset (20 s DCT Head 109kV; Siemens) was used for detection of intracranial hemorrhage and ischemic lesions. Door to FDCT time, defined as the time difference between the FDCT acquisition and patient registration, was 8 min. After intravenous injection of 60 ml contrast media with a power injector (injection rate 5 ml/s) followed by 60 ml of saline chaser, a standard biphasic FDCTA (2 × 10 s DCT Head 70kV; Siemens) image was acquired. We timed the first phase of the mpFDCTA after a bolus-tracking digital subtraction angiography (Fig. 1c), while the second phase was acquired automatically with a delay of 5 s. Raw data are automatically and quickly (~20 s) transferred and reconstructed on a commercially available workstation (syngo X workplace; Siemens). The FDCT images (Fig. 1a, b) were quickly evaluated and an intracranial bleeding was ruled out. The brain parenchyma showed only few early ischemic changes (Fig. 1a). We applied the Alberta stroke program early CT score (ASPECTS) and rated the FDCT images with 7 points. An occlusion of the M1 segment of the right middle cerebral artery (MCA) was identified on the early phase of the mpFDCTA (Fig. 1d). An occlusion or relevant stenosis of the proximal internal carotid artery could be excluded with the same acquisition. The second phase of the mpFDCTA scan was used to additionally evaluate late collaterals (Fig. 1f). According to institutional standards, all patients with an LAO are treated with thrombectomy within the first 6 h after symptom onset, regardless of the ASPECTS. Systemic application of recombinant tissue plasminogen activator was initiated and EVT was performed with the patient under conscious sedation. An 8F sheath introducer was placed in the right femoral artery 15 min after acquisition of the FDCT, resulting in a door to groin time of 23 min. The total times from hospital admission and from groin puncture to reperfusion (Fig. 1h) were 59 min and 36 min, respectively.

## Discussion

In this case report, we demonstrate to the best of our knowledge the first mothership stroke patient triaged and treated in the angio-suite with a one stop management. The ability to image and treat stroke patients in the same room without the need for transportation between modalities resulted in a door to groin time of 23 min and a door to reperfusion time of 59 min. Both times are below the recently propagated ideal target intervals of 60 min and 90 min for door to groin and door to reperfusion, respectively [5]. On the contrary, only a low proportion (13%) of patients were treated within the ideal target intervals in a recent meta-analysis of randomized trials [3].

Regarding the image quality of FDCT, it has been shown that the latest generation of FDCT is of sufficient diagnostic value, compared with multidetector CT, for the detection of intracerebral hemorrhage, albeit with a limited sensitivity on a voxel-based assessment level [6, 7]. The image quality of the latest generation of flat detector equipped angiography suite (Artis Q; Siemens) is not only sufficient to distinguish bleeding, but seems feasible in the detection of supratentorial ischemic changes (Fig. 1a; [6]). Occluded vessels can be detected with the arterial phase of FDCTA, while the addition of a venous phase allows evaluation of late cerebral collaterals. Collateral status, even when evaluated on single-phase CTA images, is a very good predictor of both patient outcome and treatment effect after EVT [8]. Accordingly, FDCT and mpFDCTA images seem to be sufficient to decide whether a patient with acute stroke is eligible for endovascular thrombectomy; however, all images in this case report were evaluated by an experienced neuroradiologist (>5 years experience with FDCT) and so a larger study is needed to evaluate the feasibility of FDCT in acute stroke diagnostics. Such a study should put special emphasis on the effect of one stop management on intrahospital time and potentially on neurological outcome but also on the safety of FDCT in the diagnosis of hemorrhagic stroke.

In summary, direct transport of patients with symptoms suggestive of LAO to the angiography suite with the combination of imaging and treatment as a one stop management approach seems to be a promising tool to optimize the management of stroke patients. Faster supply of treatment and short time intervals from hospital admission to reperfusion are expected to positively influence clinical outcome.
